# Three-Dimensional Empirical AoA Localization Technique for Indoor Applications

**DOI:** 10.3390/s19245544

**Published:** 2019-12-15

**Authors:** Abdallah Alma’aitah, Baha’ Alsaify, Raed Bani-Hani

**Affiliations:** Department of Network Engineering and Security, Jordan University of Science and Technology, P.O. Box 3030, Irbid 22110, Jordan; baalsaify@just.edu.jo (B.A.); Rbanihani@just.edu.jo (R.B.-H.)

**Keywords:** localization, angle of arrival, antenna array, non-linear error, IoT, three-dimensional localization

## Abstract

Small and pervasive devices have been increasingly used to identify and track objects automatically. Consequently, several low-cost localization schemes have been proposed in the literature based on angle of arrival (AoA), time difference of arrival (TDoA), received signal strength indicator (RSSI) or their combinations. In this paper, we propose a three-dimensional empirical AoA localization (TDEAL) technique for battery-powered devices. The proposed technique processes the AoA measurements at fixed reader nodes to estimate the locations of the tags. The proposed technique provides localization accuracy that mitigates non-linear empirical errors in AoA measurements. We utilize two omni-directional antenna arrays at each fixed reader node to estimate the location vector. With multiple location estimations from different fixed reader nodes, each estimated location is assigned a weight that is inversely proportional to the AoA phase-difference error. Furthermore, the actual AoA parabolic formula of the location is approximated to a cone to simplify the location calculation process. The proposed localization technique has a low hardware cost, low computational requirements, and precise location estimates. Based on the performance evaluation, significant location accuracy is achieved by TDEAL; where, for instance, an average error margin of less than 13 cm is achieved using 10 readers in an area of 10 m× 10 m. TDEAL can be utilized to provide reference points when integrated with a relative (e.g., inertial navigation systems) localization systems.

## 1. Introduction

Non-satellite based localization is a fundamental basis for a multitude of Internet of Things (IoT) smart environment applications. Since IoT device fabric comprises low power and small footprint devices (e.g., Radio Frequency Identification (RFID) tags, Zigbee nodes, Bluetooth tags, etc.), such devices, with their typical long lifetime, are ideal for indoor radio frequency (RF) based localization. In literature, several techniques have been proposed aiming at low infrastructure cost, high accuracy, and low computational complexity. Such techniques can broadly be categorized into range- and range-free based localization.

In range-based techniques, time difference of arrival (TDoA), time of arrival (ToA), angle of arrival (AoA), and received signal strength indicator (RSSI) are dominant [1,2,3,4,5,6,7]. TDoA and ToA are sensitive to timing errors; thus, accurate time synchronization between the readers (receivers) of these IoT devices (senders) is crucial. Moreover, line-of-sight (LOS) requirements are needed for accurate estimation of the target location. RSSI techniques, on the other hand, estimate the range between the sender and the receiver based on a log-normal distance path loss model (with or without path loss exponent updates) [8]. Nonetheless, indoor RSSI is sensitive to noise and the target location itself as the target signal experience fluctuations in the path loss exponent within the same environment, hence, RSSI generally provides a rough estimation of the object’s location. The second primary range-based technique is AoA; the angle at which the signal arrives at the antenna of the receiver is utilized to estimate the sender’s location. Using two (or more) AoAs with known receiver absolute position, the absolute coordinate of the sender can be determined by any multi-lateration method. The main challenges in AoA based techniques are the extra hardware at the receiver to estimate AoAs and the non-linear location-dependent error in the AoA [9].

By contrast, range-free techniques are mainly based on fingerprint matching [10,11,12,13,14,15,16]. Fingerprint matching is a classification technique that utilizes deep-learning techniques to recognize and match the unique signal features form the target device, at the given location, to a database of previously recorded data [14]. Even though the matched location is returned with relatively high precision, fingerprinting is time-consuming and sensitive to any changes in the environment, which requires continuous database maintenance.

In this paper, three-dimensional empirical AoA localization (TDEAL) is proposed. The TDEAL technique localizes a sender (hereafter referred to as a tag) in 3D space with respect to a fixed reference point (hereafter referred to as a reader). The scheme utilizes the robustness of AoA and the careful placement of readers at well-known and specified locations. The readers with antenna arrays are placed on the sidewalls of the localization area so that radio signals from the target are in the LOS of the readers. The collected AoAs from all readers are reported to a central database to apply the proposed method in localizing the target device at that given time. No modification on the tag is needed; however, two-antenna arrays are added to each reader to measure AoA.

The contributions of this work are:

1. The proposed TDEAL technique utilizes realistic (empirical) non-linear AoA in the calculation of the tag location.
2. The proposed technique employs a weighted-sum that is inversely proportional to AoA phase-difference error to achieve higher location accuracy. The weight assignment in TDEAL promotes location estimations with low AoA error.
3. The estimation of the tag’s location is approximated to cone equations (based on the linear slope of the cone) instead of hyperbola quadratic equations. This simplifies the calculation without sacrificing the accuracy of the estimated location.

The rest of the paper is organized as follows. In Section 2, the state of the art techniques in 3D localization are introduced. In Section 3, the system model and the fundamentals of 3D AoA based localization are described. The TDEAL technique is proposed in Section 4 including location vector by one reader, location estimation based on two readers measurements, and weight assignment and algorithm. In Section 5, TDEAL is evaluated under different numbers of readers with tag at various locations and heights. The paper is concluded in Section 6 while providing future directions.

## 2. Related Work

Several node localization schemes have been proposed in the literature. The primal aim was to provide an estimate of the two or three-dimensional location in reference to an absolute reference point. In [17], Mao et al. studied the different techniques to determine the location of a specific node in space. They categorized the different methods and algorithms into two main categories; range-based and range-free approaches. Range-free methods produce coarse location results when compared with the range-based schemes [18]. In addition, range-free approaches require extensive deployment planning and surveying and the localization error in these approaches are prone to changes in the environment [18]. Range-based approaches, on the other hand, are divided mainly into ToA/ TDoA, RSSI, and AoA.

For TDoA, researchers attempt to determine the location of an object based on the signal’s ToA from different sources. Subtracting the ToA of the signals will eliminate the need for determining the time at which the signal was generated thus eliminating the need for accurate clock synchronization at the objects to be localized. However, the clock synchronization issue will remain a critical part of the objects performing the localization process. In [19], the researchers compared ToA with TDoA in terms of the localization error. Although the TDoA approach sounds much better than the ToA approach, the simulations performed showed no difference in the localization performance. Other examples that utilize these techniques can be found in [20,21].

For RSSI, much work had been done on estimating the tag’s location and distance based on RSSI measurements. In general, to determine the location of an object based on the received signal strength, you mainly need two pieces of information: the transmitter′s location, and the signal strength at the transmitter. In [22], the researchers investigated a relationship between the radar cross section (RCS) and the maximum read range to estimate the target location. In [23], instead of determining the location of RFID tags, it attempts to determine the location of an RFID reader by analyzing the received radio signals transmitted from a distributed set of passive tags. The researchers divided their work into three stages; first, they study the environment to determine the effect it has on the RSSI measurements. Next, they define an estimate of the reader’s location. Finally, they developed a system to track a RFID reader taking into consideration its location estimate and the velocity it moves in with an error of 31 cm. Other works on RSSI and its usage for localization can be found in [24,25,26,27].

In AoA-based schemes, clock synchronization among multiple nodes is not needed. Amundson et al. [15] proposed a localization system using radio interferometric AoA for wireless sensor nodes. However, the scheme is not immune to multipath. Alsalih et al. [28] provide a combination of the RSSI localization method and localization based on AoA which is called angle of arrival cluster forming (ACF). Tomic et al. [29], used both the RSSI value and the AoA to locate and track a wireless emitting mobile device. Wielandt et al. [30] proposed an AoA scheme in which spatial smoothing of the reference vectors is applied. This approach was empirically evaluated in LOS and non-LOS (NLOS) scenarios. Note that AoA in the literature is usually combined with other range- or range-free mechanisms to increase localization accuracy. Other range-based methods that comprise AoA can be found in [31,32]. In our proposed TDEAL technique, we estimate the tag locations in three-dimensional space while accounting for the non-linearity of the empirical AoA error profile.

## 3. System Model

### 3.1. System Components 

**Figure 1:**
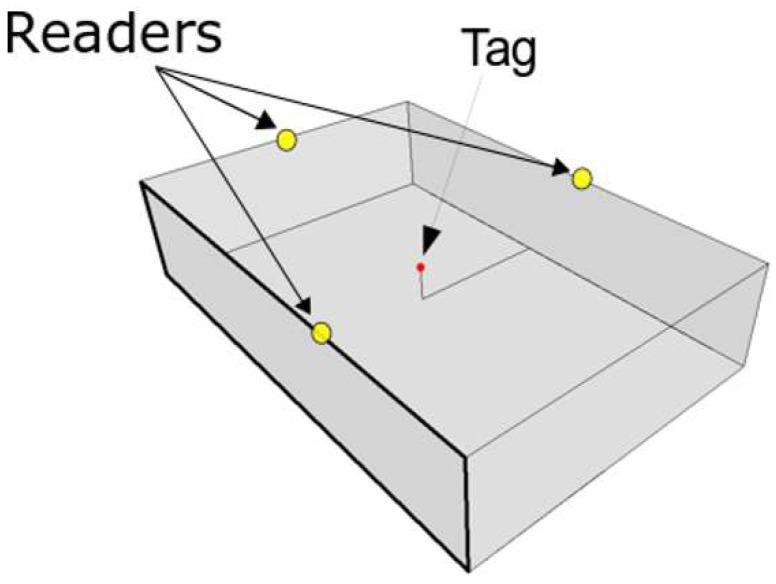
An example of a 3-reader placement with one tag model in the localization area.

We employ *N* readers to determine the 3D relative positions of *K* IoT devices tags. Let $R_{1}\ldots R_{N}$ denote the *N* readers and $T_{1}\ldots .T_{K}$ denote the *K* tags. The positions of the readers are pre-determined and placed at the sidewalls of the area at which the tags are to be localized. This is to ensure clear LOS paths or LOS paths that are not blocked by metallic materials. If the readers are placed at lower elevation, higher number of readers will be needed to guarantee LOS with the tags. All readers in our technique have the same elevation (i.e., the ceiling of the localization area), and consequently, the elevation of the tags is never higher than the readers’ elevation. An example of this model is illustrated in Figure 1 with three readers and one tag.

**Figure 2:**
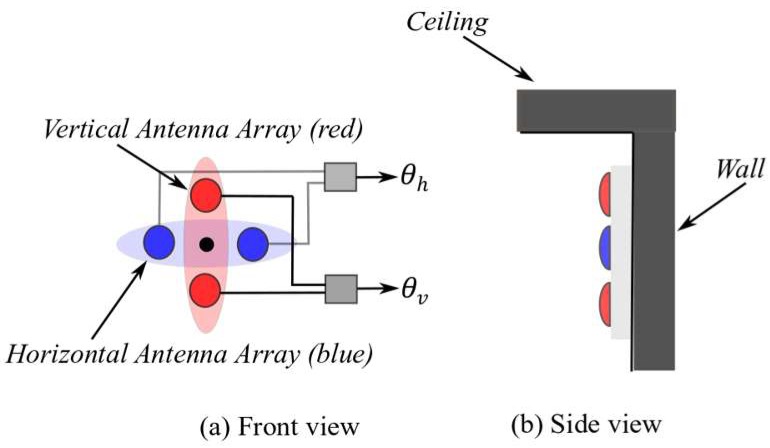
(**a**) Front view of two antenna arrays at the reader; (**b**) side view showing placement on the sidewall.

Each tag uses an omni-directional antenna to broadcast its signals, while a reader receives the signals from the tags with vertical and horizontal antenna arrays. Each array consists of two antennas for AoA measurement. A reader with two antenna arrays is depicted in Figure 2, one array to measure the vertical AoA ($\theta_{v}$) and the other to measure the horizontal AoA ($\theta_{h}$). Please note that $\theta_{v}$ and $\theta_{h}$ are not the azimuth (α) and elevation (β) angles in a given 3D space. However, α and β are calculated from horizontal and vertical AoA angles as will be discussed in Section 4.

The readers are assumed to be placed at a known location in the common orthogonal coordinate system (x,y,z) where *x*-*y* plane is the horizontal plane, and *z* is the height (vertical elevation). The location of any given reader $R_{n}\;$is denoted by $\left(x_{n},\;y_{n},\;z_{n}\right).\;$The location of the tag is similarly denoted by $\left(x_{k},\;y_{k},\;z_{k}\right)$. It is worth mentioning that the reader coordinates are representing the center of the four antennas (black circle), as shown in Figure 2a.

### 3.2. Angle of Arrival (AoA) Calculation by Two Antennas

**Figure 3:**
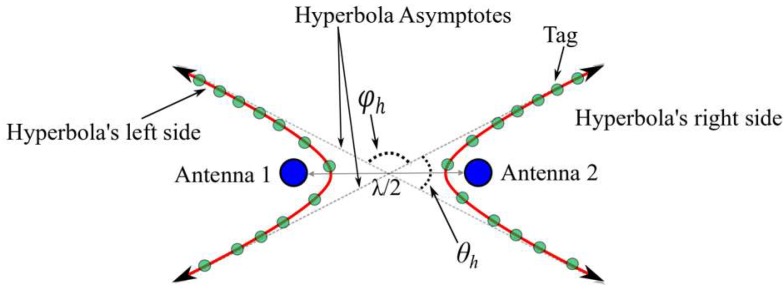
The hyperbolas (red lines) at which all tags will have the same phase difference between antenna 1 and antenna 2.

To estimate the angle of arrival of the tag’s signal, an antenna array composed of two individual antenna elements are used. The two antenna elements are separated by a distance of $\frac{\lambda }{2}$ m, where λ is the wavelength of the communication channel frequency. The array is aligned alongside the horizontal (within the *x*-*y* plane) or the vertical (along the *z*-axis). The phase difference φ at two antennas in a given array is measured by $\frac{2\pi \left(d_{1}-d_{2}\right)}{\lambda }$, where *d_1_* and *d_2_* are the distances between the tag and the first and second antenna in the array, respectively. In Figure 3, an example of a horizontal antenna array is provided. The phase difference $\varphi_{h}$ is the same when the tag (represented by the opaque green circles) is at any point of the red line. The red line represents a hyperbola with its foci antenna 1 and antenna 2. Note that the angle between the hyperbola’s asymptotes is denoted by $\theta_{h}\;=\;\pi -\varphi_{h}$.

**Figure 4:**
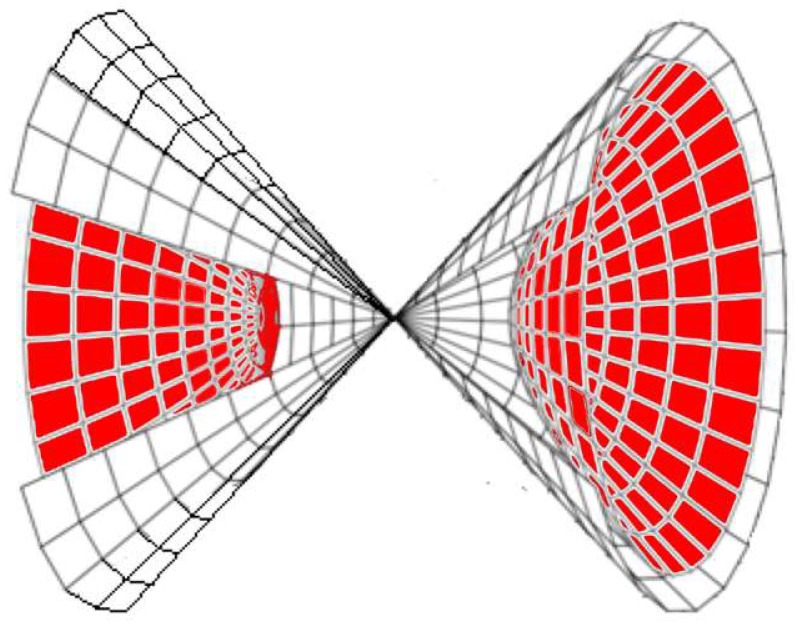
Three-dimensional (3D) two-sheeted hyperbola around a horizontal antenna array (in red). The asymptotes provide a two cones around the two-sheeted hyperbola.

To adapt the above relation between the tag location and the horizontal angle to a 3D space, for every measured $\theta_{h}$ there exists a 3D two-sheeted hyperbola on which the tag can reside as shown in Figure 4 by the red two sheets (which is the rotation of the 2D hyperbola and its asymptotes in Figure 3). The outer cones around the two red hyperbola sheets in Figure 4 are the asymptotes sheets of that given hyperbola. For any given reader$\;R_{n}$ with a known position at $\left(x_{n},\;y_{n},\;z_{n}\right)$, the two focal points of any hyperbola are the two antennas, as shown in Figure 3, with a difference of$\;2c\;=\;\frac{\lambda }{2}\;$ The equation of the resulting two-sheeted hyperbola for a tag at a location $\left(x_{k},\;y_{k},\;z_{k}\right)$ is given by:

(1) $$-{\left(x_{k}-x_{n}\right)}^{2}-{\left(y_{k}-y_{n}\right)}^{2}+\;{\left(\frac{z_{k}-z_{n}}{m}\right)}^{2}\;=\;1,$$

where $m\;=\;\frac{a}{b},\;$ a = $\frac{\lambda \theta_{h}}{4\pi }$ is the main vertex of the hyperbola, and$\;b\;=\;\sqrt{c^{2}-a^{2}}$ is the co-vertex. Since any location on the two-sheeted hyperbola satisfies Equation (1), more than one antenna array is needed to estimate a unique tag location.

## 4. Three-Dimensional Empirical AoA Localization (TDEAL)

In this section, the three-dimensional empirical AoA localization (TDEAL) is presented. The solution based on one reader is first introduced. The effect of the error in AoA is then illustrated, followed by tag location estimation two readers. The weighted average of the estimated location of a given tag is defined to mitigate location estimates based on high AoA errors. Finally, TDEAL location estimation algorithm is given to summarize the technique steps.

### 4.1. Location Solution Based on $\theta_{v}$ and $\theta_{h}$ AoA Readings

In our location estimation algorithm, we reduce the complexity of the hyperbola equations by using a cone formed by the asymptotes of that hyperbola. This is justifiable as the distance between the target tag $T_{k}\;$and any reader $R_{n}$ is typically much more than half of the wavelength (λ/2), and the hyperbola at such distances approaches its asymptotes (cones in three-dimensional space) rapidly. For instance, at 2.45 GHz channel frequency, λ/2 is approximately 0.0612 m. If a tag is placed at one meter distance from the reader, the difference between the cone and the hyperbola sheets is less than 0.0023 m. Furthermore, the gap is shrinking dramatically as the tag goes further from the reader. Therefore, Equation (1) is replaced by the equation of the cone given by the measured $\theta_{v}$ at a reader $R_{n}$ by a tag $T_{k}$ which is:

(2) $$-{\left(x_{k}-x_{n}\right)}^{2}-{\left(y_{k}-y_{n}\right)}^{2}+\;{\left(\frac{z_{k}-z_{n}}{m_{v}}\right)}^{2}\;=\;0.$$

Note that $m_{v}$ is the slope of the asymptote of the hyperbola formed at that $\theta_{v}$ when the two antennas are placed parallel to the *z*-axis. Similarly, for the horizontal antenna array in which the antennas are placed alongside the *x*-axis to measure $\theta_{h}$, Equation (2) is replaced by:

(3) $$-{\left(z_{k}-z_{n}\right)}^{2}-{\left(y_{k}-y_{n}\right)}^{2}+\;{\left(\frac{x_{k}-x_{n}}{m_{h}}\right)}^{2}\;=\;0.$$

**Figure 5:**
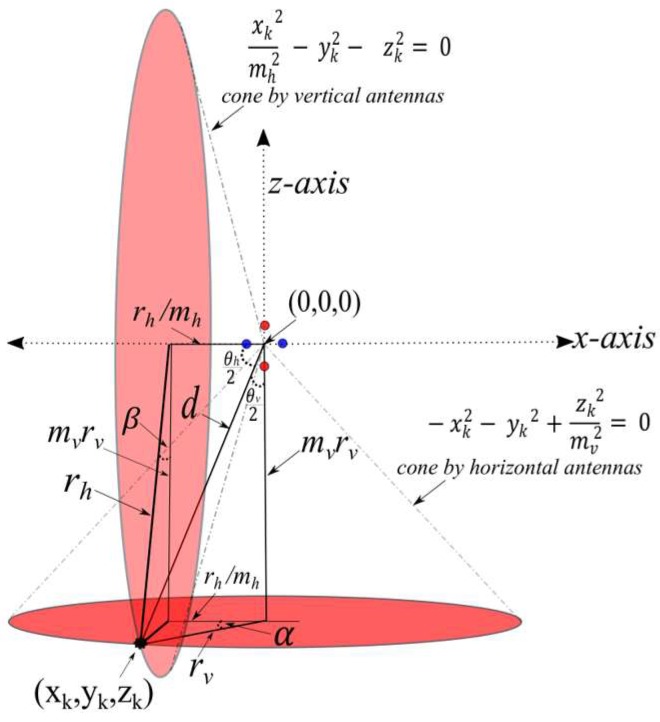
Azimuth and elevation angles relation to angle of arrival (AoA) measurements from horizontal and vertical antennas.

As illustrated in Figure 5, for a given tag $T_{k}\;$at an unknown location$\;\left(x_{k},\;y_{k},\;z_{k}\right)$, the vertical (red circles) and horizontal (blue circles) antenna arrays of the reader $R_{n}$ will produce the angles $\theta_{v}$ and$\;\theta_{h}$, respectively. Without loss of generality, if $R_{n}$ coordinates are at the origin of the *x*, *y*, *z* space (i.e., (0,0,0)), then the two equations formed by the phase difference measurements $\theta_{v}$ and$\;\theta_{h}$ will be:

$$-x_{k}^{2}-y_{k}{}^{2}+\;{\left(\frac{z_{k}}{m_{v}}\right)}^{2}\;=\;0\;\mathrm{and}\;{\left(\frac{x_{k}}{m_{h}}\right)}^{2}-y_{k}^{2}-\;z_{k}^{2}\;=\;0$$

Figure 5 illustrates the intersection line labeled by d between the two cone equations. We will shortly represent all possible intersection (other than d) between the two cone equations. This illustration will show the relation between phase difference measurements $\theta_{v}$ and$\;\theta_{h}$ and azimuth and elevation angles α and β, respectively. Note that,$\;m_{v}\;=\;\frac{1}{\tan\left(\frac{\theta_{v}}{2}\right)}$ and $m_{h}$ $=\;tan\frac{\theta_{h}}{2}$ and the magnitude of line d is: $∥d∥\;=\;r_{v}\sqrt{1+m_{v}^{2}}\;=\;r_{h}\sqrt{1+1/m_{h}^{2}}.$ Therefore, $r_{h}=r_{v}\sqrt{1+m_{v}^{2}}/\sqrt{1+1/m_{h}^{2}}\;$. Let $\xi_{v}\;=\sqrt{1+m_{v}^{2}}$, and $\xi_{h}\;=\;\sqrt{1+1/m_{h}^{2}}$; then as $\cos\alpha \;=\;\frac{r_{h}}{r_{v}m_{h}}\;=\;\frac{\sqrt{1+m_{v}^{2}\;}}{m_{h}\sqrt{1+\frac{1}{m_{h}^{2}}}}\;=\;\frac{\xi_{v}}{m_{h}\xi_{h}},\;$ The azimuth angle α is $\cos^{-1}\left(\frac{\xi_{v}}{m_{h}\xi_{h}}\right)$.

Similarly, as $\cos\beta \;=\;\frac{r_{v}m_{v}}{m_{h}}\;=\frac{m_{v}\;\sqrt{1+1/m_{h}^{2}\;}}{\sqrt{1+m_{v}^{2}}}\;=\;m_{v}\xi_{h}/\xi_{v},\;$the elevation angle β is $\cos^{-1}\left(\frac{m_{v}\xi_{h}}{\xi_{v}}\right)$. Any tag at the vector $\vec{d}$ satisfies both azimuth and elevation angles α and β. Even though the intersection of the vertical and horizontal cones do not produce a unique coordinate of a given tag, it reduces the possibilities from a surface of a cone (by one antenna array) to vectors that beam from the reader coordinates (intersection of two cones from two antenna arrays).

**Figure 6:**
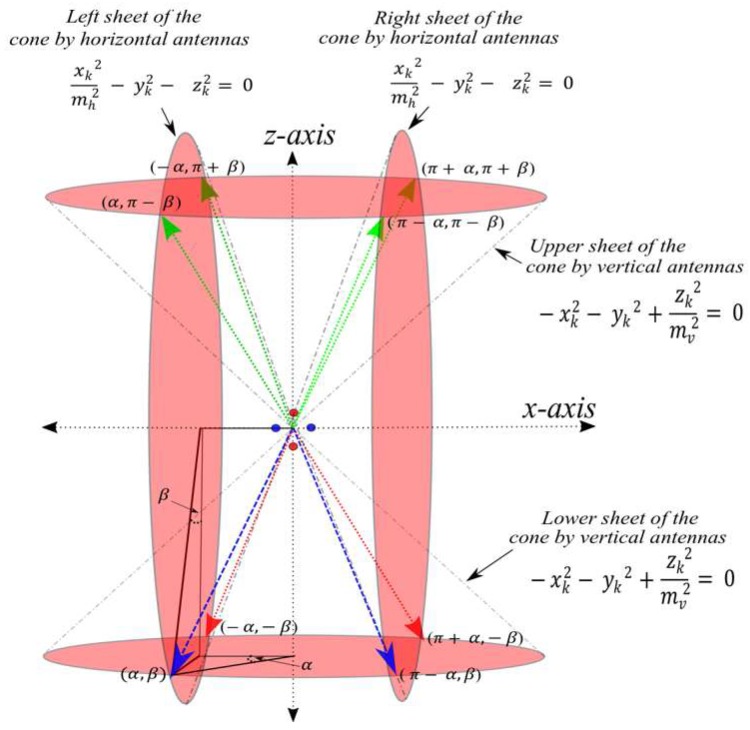
Eight vectors beaming from the center point between the two antenna arrays.

Note that the two cone equations represented in Figure 5 not only intersect in the vector $\vec{d}$, but in mirrored versions of the vector $\vec{d}$; resulting in 8 vectors beaming from the reader coordinates, as shown in Figure 6. The vectors beam at the angles (α,β), ( π−α, β), (π+α,−β), (−α,−β), (α,π−β), ( π−α, π−β), (π+α,π+β), and (−α,π+β). To eliminate a group of the vectors (i.e., to have their solution out of the localization area), we placed the readers at an elevation that is higher than the tags height (i.e., on the side of the wall or at the ceiling edge); this placement excludes six out of the eight vectors. Four vectors will be eliminated as they are above the wall (green vectors in Figure 6), and the other two vectors with angles (−α,−β) and (π+α,−β) will be behind the wall (red vectors in Figure 6). Therefore, the location solution is limited by the remaining two vectors in Figure 6 with angles (α,β) and ( π−α, β) (blue colored vectors in Figure 6). To reduce the vector solutions to a point, another reader/readers should be placed at non-collinear locations as will be discussed in Section 4.3.

### 4.2. Location Solution Based on Non-Linear Angle Errors $\varepsilon_{v}$ and $\varepsilon_{h}$

The phase error plays a significant role in the accuracy of the estimated target location. In AoA detectors, the accuracy of the estimated tag location depends on the location itself; hence, the non-linearity in phase error [33]. To consider the effect of the vertical and horizontal phase errors $\varepsilon_{v}$ and $\varepsilon_{h}$ at a given reader$\;R_{n}$, the vertical and horizontal slopes, $m_{v}^{\pm \varepsilon }\;$ and $m_{h}^{\pm \varepsilon }$, will become ${\left(\tan\left(\frac{\theta_{v}\pm \varepsilon_{v}}{2}\right)\right)}^{-1}$ and $\;\tan\left(\frac{\theta_{h}\pm \varepsilon_{h}}{2}\right)$, respectively.

**Figure 7:**
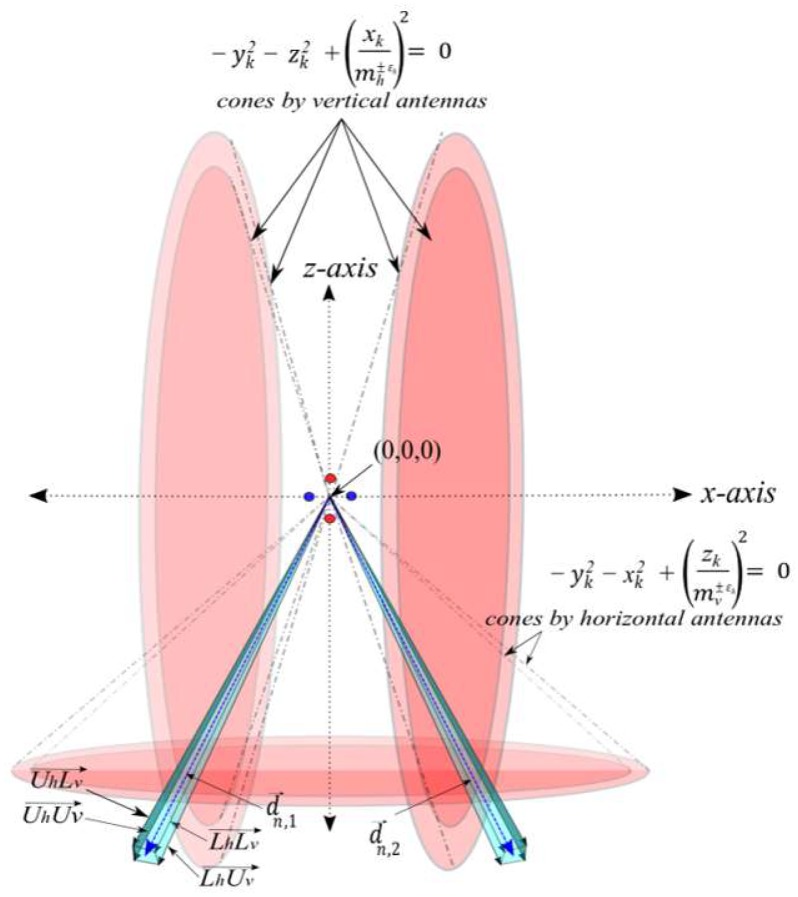
Interstation vectors of two antenna arrays with error margins.

By introducing the error ε, each antenna array will have two equations comprising the upper and lower bounds of ε. Therefore, estimating the tag’s location vectors are no longer the solution of two quadratic polynomial equations. Two cones from the vertical antenna arrays will intersect with two cones from the horizontal array. The resultant intersection of the four cones is a rectangular horn-like volume bounded by the intersections of the following four equations $-x_{k}^{2}-y_{k}{}^{2}+\;{\left(\frac{z_{k}}{{\left(\tan\left(\frac{\theta_{v}\pm \varepsilon_{v}}{2}\right)\right)}^{-1}}\right)}^{2}\;=\;0$ and ${\left(\frac{x_{k}}{\tan\left(\frac{\theta_{h}\pm \varepsilon_{h}}{2}\right)}\right)}^{2}-y_{k}^{2}-\;z_{k}^{2}\;=\;0$. The intersection volume is illustrated in Figure 7.

The resultant two solution volumes in Figure 7 deviate in proportion to $\varepsilon_{v}$ and $\varepsilon_{h}$. The four cones now intersect in four vectors instead of a single vector as in the case where no error around $\theta_{v}$ and$\;\theta_{h}$. The vectors are denoted by:

1. ($\vec{U_{h}U_{v}}$): The possible intersection between the cone with$\;m_{h}^{+\varepsilon }$ and the cone with$\;m_{v}^{+\varepsilon }$.
2. ($\vec{U_{h}L_{v}}$): The possible intersection between the cone with$\;m_{h}^{+\varepsilon }$ and the cone with $\;m_{v}^{-\varepsilon }$.
3. ($\vec{L_{h}U_{v}}$): The possible intersection between the cone with $\;m_{h}^{-\varepsilon }$ and the cone with$\;m_{v}^{+\varepsilon }$.
4. ($\vec{L_{h}L_{v}}$): The possible intersection between the cone with$\;m_{h}^{-\varepsilon }$ and the cone with $\;m_{v}^{-\varepsilon }$.

The k (where 1≤k≤4) possible intersection vectors are averaged to provide a single vector representing the estimated location vector by a reader $R_{n}$. The average vector is denoted by ${\vec{d}}_{n,1}$ and defined by angles $\beta_{n,1}\;=\;\frac{\sum_{1}^{k}\beta_{k}}{k}$ and$\;\alpha_{n,1}\;=\;\frac{\sum_{1}^{k}\alpha_{k}}{k}$ (left blue vector in Figure 7). By symmetry, $\beta_{n,2}$ and $\alpha_{n,2}$ angles of the second estimated location vector ${\vec{d}}_{n,2}$ (right blue vector in Figure 7) are obtained.

### 4.3. Location Estimation by Two Readers

In this subsection, we estimate the location of the tag under empirical AoA error model. Tag location can be estimated to a given point if two vectors from two readers intersect. Given two readers $R_{n}$ and $R_{m}$, $R_{n}$ has ${\vec{d}}_{n,1}$ and ${\vec{d}}_{n,2}$ as a solution vectors, and $R_{m}$ will have the solution vectors ${\vec{d}}_{m,1}$ and ${\vec{d}}_{m,2}$. If $R_{n}\;$and $R_{m}\;$are non-collinear (i.e., the *z* and *y*, or *z* and *x* of $R_{n}\;$and $R_{m}$ coordinates are not equal), only one vector from $R_{n}$ will intersect with one vector from$\;R_{m}$. Otherwise, if the two readers are collinear (i.e., $R_{n}\;$and $R_{m}$ are facing each other), the vectors ${\vec{d}}_{n,1}$ and ${\vec{d}}_{n,2}$ which are symmetrical around the vertical antennas will intersect at equal distances from$\;{\vec{d}}_{m,1}$ and${\vec{\;d}}_{m,2}$, respectively. Such collinearity will cause two solutions instead of one solution, which is not desired. 

Note that the solution vectors$\;{\vec{d}}_{n,1}$, ${\vec{d}}_{n,2}$, ${\vec{d}}_{m,1}$, and ${\vec{d}}_{m,2}$ inherit the errors in the vertical and horizontal AoAs. Therefore, two of the vectors *may* or *may not* intersect due to deviation caused by angle error. In case of an intersection, the intersection coordinates are considered the estimated tag location and denoted by ${\hat{F}}_{n,m}$. In case of no intersection, the midpoint of the shorted orthogonal distance between the two vectors is considered the estimated location of the tag. Given the centers of the readers $R_{n}$ and $R_{m}$ as ($x_{n},\;y_{n},\;z_{n}$) and $(x_{m},\;y_{m},\;z_{m}),$ respectively, and the solution vectors:

$${\vec{d}}_{n,1}\;=\;\left(\begin{array}{c} i_{n,1}\\ \begin{array}{c} j_{n,1}\\ k_{n,1} \end{array} \end{array}\right),\;{\vec{d}}_{n,2}\;=\;\left(\begin{array}{c} i_{n,2}\\ \begin{array}{c} j_{n,2}\\ k_{n,2} \end{array} \end{array}\right),\;{\vec{d}}_{m,1}\;=\;\left(\begin{array}{c} i_{m,1}\\ \begin{array}{c} j_{m,1}\\ k_{m,1} \end{array} \end{array}\right),\;\mathrm{and}\;{\vec{d}}_{m,2}\;=\;\left(\begin{array}{c} i_{m,2}\\ \begin{array}{c} j_{m,2}\\ k_{m,2} \end{array} \end{array}\right),$$

where i = sinαcosβ, j = sinβcosα, and k = sinβ. The shortest distance between ${\vec{d}}_{n,1}$ and ${\vec{d}}_{m,1}$ can be measured by assuming a point $P_{1,1}$ on ${\vec{d}}_{n,1}$ and $Q_{1,1}$ on ${\vec{d}}_{m,1}$. The coordinates of the two points can be written as: $P_{1,1}\;=\;\left(\begin{array}{c} x_{n}+ti_{n,1}\\ \begin{array}{c} y_{n}+tj_{n,1}\\ z_{n}+tk_{n,1} \end{array} \end{array}\right),$ and $Q_{1,1}\;=\;\left(\begin{array}{c} x_{m}+li_{m,1}\\ \begin{array}{c} y_{m}+lj_{m,1}\\ z_{m}+lk_{m,1} \end{array} \end{array}\right)$.

Therefore, the vector$\;\vec{PQ_{1,1}}\;=\left(\begin{array}{c} x_{n}+ti_{n,1}-x_{m}-li_{m,1}\\ \begin{array}{c} y_{n}+tj_{n,1}-y_{m}-lj_{m,1}\\ z_{n}+tk_{n,1}-z_{m}-lk_{m,1} \end{array} \end{array}\right)$. If there are values of t and l that makes$\vec{\;PQ_{1,1}}\;=\;0$, the two lines intersects, otherwise, we have to find the values of t and l that satisfy: $\vec{PQ_{1,1}}\cdot {\vec{d}}_{n,1}\;=\;\left(\begin{array}{c} x_{n}+ti_{n,1}-x_{m}-li_{m,1}\\ \begin{array}{c} y_{n}+tj_{n,1}-y_{m}-lj_{m,1}\\ z_{n}+tk_{n,1}-z_{m}-lk_{m,1} \end{array} \end{array}\right)\cdot \left(\begin{array}{c} i_{n,1}\\ \begin{array}{c} j_{n,1}\\ k_{n,1} \end{array} \end{array}\right)\;=\;0$, and $\vec{PQ_{1,1}}\cdot {\vec{d}}_{m,1}\;=\;\left(\begin{array}{c} x_{n}+ti_{n,1}-x_{m}-li_{m,1}\\ \begin{array}{c} y_{n}+tj_{n,1}-y_{m}-lj_{m,1}\\ z_{n}+tk_{n,1}-z_{m}-lk_{m,1} \end{array} \end{array}\right)\cdot \left(\begin{array}{c} i_{m,1}\\ \begin{array}{c} j_{m,1}\\ k_{m,1} \end{array} \end{array}\right)=0$.

The above two scalar products will provide two linear equations to solve t and l and, therefore, solve the points $P_{1,1}$ and $Q_{1,1}$. The estimated tag location by vectors ${\vec{d}}_{n,1}$ and ${\vec{d}}_{m,1}$ will be:

$$\hat{F_{n,m}^{1,1}}\;=\;P_{1,1}+\vec{PQ_{1,1}}\frac{|{\vec{PQ}}_{1,1}|}{2}\;=\;Q_{1,1}-\vec{PQ_{1,1}}\frac{|\vec{PQ_{1,1}}|}{2}.$$

The same applies to the remaining three solution vectors pairs (${\vec{d}}_{n,1}$, ${\vec{d}}_{m,2})$, (${\vec{d}}_{n,2}$, ${\vec{d}}_{m,1}),$ and (${\vec{d}}_{n,2}$, ${\vec{d}}_{m,2})$. ${\hat{F}}_{n,m}$ is the midpoint of the shortest $|\vec{PQ}|$ of all solution vector pairs.

### 4.4. Angle-Weighted Location Estimation

The tag location is estimated at a single coordinate by every pair of readers. Therefore, if a tag is detected by *N* readers, then there will be $H\;=\;\left(\begin{array}{c} N\\ 2 \end{array}\right)$ estimated locations for that given tag. If there is no error in AoA measurements, then all estimated locations will be at a single point. However, the error in AoA does not only exist but also is angle-dependent [9]. In addition, even a small error in AoA measurement causes a deviation between the two cones that is proportional to the distance from the reader. Therefore, not all estimated points, of a tag $T_{k}$, ${\hat{F}}_{i,k}$ (where i∈{1,…, H}) have the same accuracy. Consequently, taking the simple average of the H estimated points from *N* readers produces a solution that similarly treats estimates based on high error margins and low error margins.

In the proposed TDEAL we assign weight w to account for the errors in the angles and the distance between the estimated location and the two readers. The weight w is designed such that the higher the phase error or the distance to the reader, the lower the weight. For any two readers $R_{n}$ and $R_{m}$ that are used to estimate the $l^{\mathrm{th}}$ location (i.e., ${\hat{F}}_{l,k}$) in the overall H estimates of a given tag $T_{k}$. The weight $w_{l,k}$ of ${\hat{F}}_{l,k}$ is equally proportional to the inverse of:

- The phase errors $\varepsilon_{v,n,k}$, $\varepsilon_{h,n,k},\;\varepsilon_{v,m,k},$ and $\varepsilon_{h,m,k}$ that are reflected the asymptotical slopes $(m_{v,n,k}^{+\varepsilon }$ and $m_{v,n,k}^{-\varepsilon }$), $(m_{h,n,k}^{+\varepsilon }$ and $m_{h,n,k}^{-\varepsilon }$), $\left(m_{v,m,k}^{+\varepsilon }\;\mathrm{and}\;m_{v,m,k}^{-\varepsilon }\right)$, and $(m_{h,n,k}^{+\varepsilon }$ and $m_{h,n,k}^{-\varepsilon }$).
- The maximum distance between the estimated location ${\hat{F}}_{l,k}$ of a tag $T_{k}\;$and the centers of either reader that are used for the estimation.

Then total difference of the asymptotical is denoted by $\Psi_{n,m,k}\equiv \Psi_{l,k}$ and is given by $\Psi_{l,k}\;=\;\left|m_{v,n,k}^{+\varepsilon }-m_{v,n,k}^{-\varepsilon }\right|$+$\left|m_{h,n,k}^{+\varepsilon }-m_{h,n,k}^{-\varepsilon }\right|+|m_{v,m,k}^{+\varepsilon }-m_{v,m,k}^{-\varepsilon }|+\left|m_{h,n,k}^{+\varepsilon }-m_{h,n,k}^{-\varepsilon }\right|$, and the normalized weight of the slope differences is defined as $\Psi_{l,k}^{\prime }\;=\;\frac{\left(\sum_{j\;=\;1}^{H}\Psi_{j,k}\right)-\;\Psi_{l,k}}{\left(H-1\right)\sum_{j\;=\;1}^{H}\Psi_{l,k}}$.

For the maximum distance between a tag $T_{k}$ and both $R_{n}$ and $R_{m}$ is denoted by $\Theta_{n,m,k}\equiv \Theta_{l,k}\;$ and defined as$\;\max\left(∥{\hat{F}}_{l,k}-R_{n}∥,∥{\hat{F}}_{l,k}-R_{m}∥\right)$. Subsequently, and the normalized weight of the maximum distance is defined as $\Theta_{l,k}^{\prime }\;=\;\frac{\left(\sum_{j\;=\;1}^{H}\Theta_{j,k}\right)-\Theta_{l,k}}{\left(H-1\right)\sum_{j\;=\;1}^{H}\Theta_{j,k}}$. Since $w_{l,k}\;$is equally proportional to both slope differences and maximum distance, $w_{l,k}\;=\;\frac{\Psi_{l,k}^{\prime }+\Theta_{l,k}^{\prime }}{2};$ and the weighted average of all H estimated locations of a tag $T_{k}$ is given by $\hat{F_{k}}=\overset{H}{\underset{i=1}{\sum }}w_{i,k}{\hat{F}}_{i,k}$. Note that if only two readers are localizing a tag $T_{k}$ (i.e., H = 1) $\hat{F_{k}}$ directly estimated as ${\hat{F}}_{1,k}$. To summarize the proposed TDEAL technique, Algorithm 1 is provided.
**Algorithm 1** Three-Dimensional Empirical AoA LocalizationInput: AoA readings at fixed reader nodes, three-dimensional location of N’ readers, phase error profile
Output: Estimated three-dimensional location $\hat{F}$ of a tag $T_{k}$
1. A tag broadcasts its unique identifiers2. N readers receive the signals from the tags (N⊆N′)3. Each reader $R_{n}$ reports the measured AoA $\theta_{h}$ and $\theta_{v}$ to a central database4. Map $\theta_{h}$ and $\theta_{v}$ to their corresponding AoA error $\varepsilon_{h}$ and $\varepsilon_{v}$5. If N≥26. For *n* = 1 to *N*7. Compute $m_{n,v}$ and $m_{n,h}$8. Compute the vectors ${\vec{d}}_{n,1}$ and ${\vec{d}}_{n,2}$9. End for10Else11Tag cannot be localized by one reader12End If13For *I* = 1 to $\left(\begin{array}{c} N\\ 2 \end{array}\right)$14Compute min $|\vec{PQ}|$15Compute estimated location ${\hat{F}}_{i,k}$16End For17For *I* = 1 to $\left(\begin{array}{c} N\\ 2 \end{array}\right)$18Compute $\Psi_{i,k}^{\prime }$, $\Theta_{i,k}^{\prime }$19Compute $w_{i,k}$20End For21Compute ${\hat{F}}_{k}$


The complexity of the above algorithm is analyzed when the tags are detected by N = N′ readers (i.e., all readers). $\theta_{h}$ and $\theta_{v}$ at each reader is directly measured and reported to the central database (i.e, O(1)). The mapping to the corresponding AoA errors $\varepsilon_{h}$ and $\varepsilon_{v}$ is done for all readers in 2*N* steps (i.e., O(N)). The same is done to calculate the cone slopes $m_{n,v}$ and $m_{n,h}$ in 2*N* steps (i.e., O(N)). The vectors for each reader, ${\vec{d}}_{n,1}$ and ${\vec{d}}_{n,2}$, are also calculated in 2*N* steps (i.e., O(N)). For each two readers combinations, $4*\left(\begin{array}{c} N\\ 2 \end{array}\right)\;=\;2N^{2}-2N$ steps are needed to compute min $|\vec{PQ}|$ (i.e., $O\left(N^{2}\right)$). $\left(\begin{array}{c} N\\ 2 \end{array}\right)\;=\;\frac{N^{2}}{2}-\frac{N}{2}$ steps are needed to the estimated location ${\hat{F}}_{i,k},\;i\in H$ (i.e., $O\left(N^{2}\right)$). Normalizing the weight of the slope differences $\Psi_{i,k}^{\prime }$, the weight of the maximum distance $\Theta_{i,k}^{\prime }$, and the combined weight $w_{i,k}$ requires $3*\left(\begin{array}{c} N\\ 2 \end{array}\right)$ steps (i.e., $O\left(N^{2}\right)$). Finally, the estimated location of the tag ${\hat{F}}_{k}$ is calculate in $\left(\begin{array}{c} N\\ 2 \end{array}\right)$ steps (i.e., $O\left(N^{2}\right)$). Therefore, the complexity of TDEAL algorithm is$\;O\left(N^{2}\right)$.

## 5. Performance Evaluation

Extensive simulations have been conducted to evaluate the performance of TDEAL. We have adopted the empirical reported AoA measurements in the datasheet of the AD8302 chip. Then we interpolate the resultant empirical measurements to simulate the effectiveness of the proposed scheme under different tag locations, reader count, and reader heights under empirical phase difference error profiles using MATLAB.

### 5.1. Angle Error Measurement 

**Figure 8:**
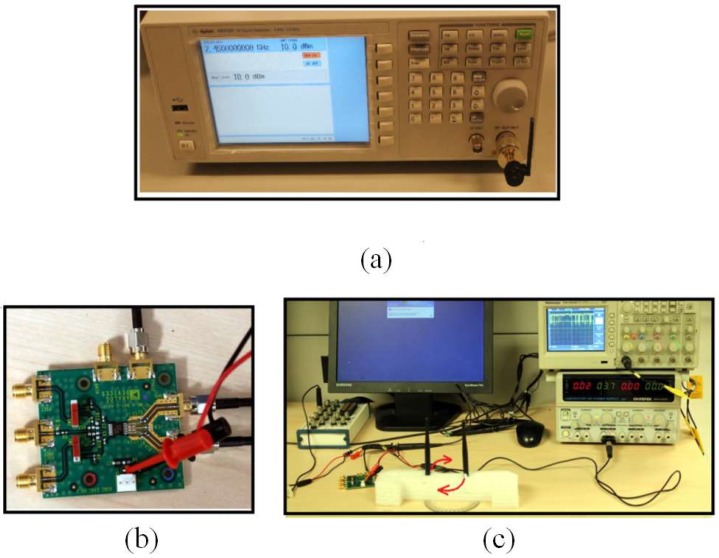
(**a**) Radio frequency (RF) signal generator. (**b**) AD8302 phase difference evaluation board. (**c**) The two antenna array connected to the phase difference module and the output is connected to the oscilloscope and data acquisition board.

**Figure 9:**
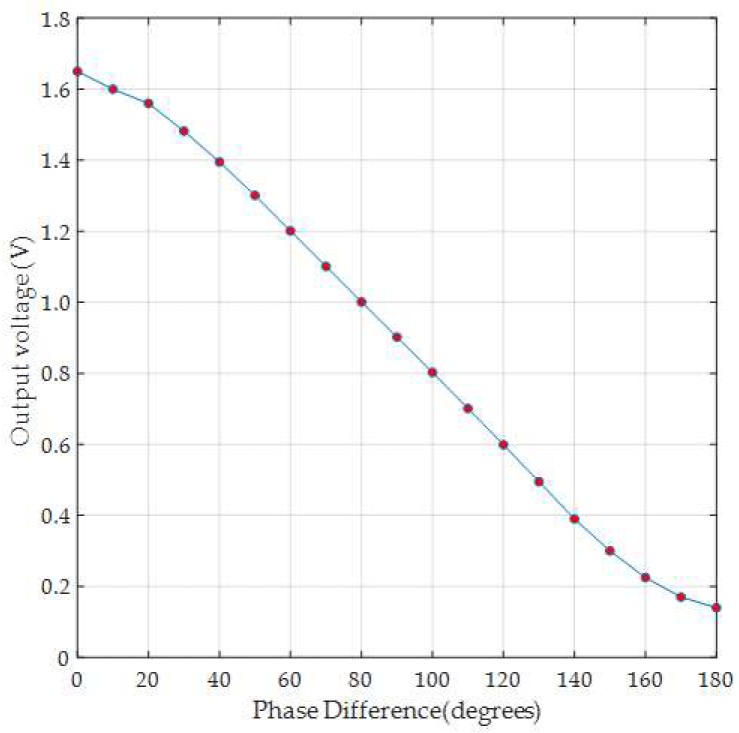
The output voltage from the phase difference module in the experiment setup in Figure 8 when rotating the antenna array from 0° to 90° (causing a phase difference from 0° to 180°).

**Figure 10:**
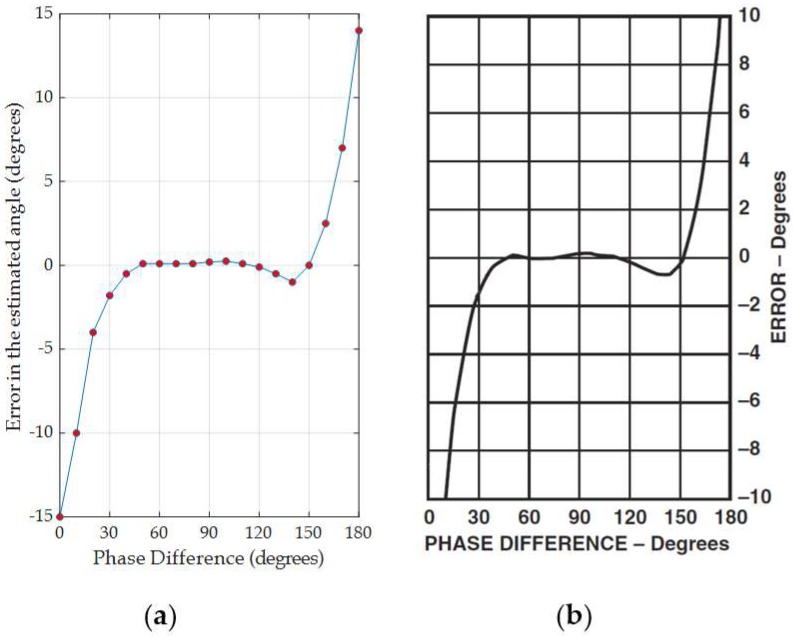
(**a**) The phase difference error (in degrees) based on the output voltage in Figure 9. (**b**) The phase difference error (in degrees) from [9] at 2.2 GHz.

In our design, we utilize a phase difference using the AD8302 chip to estimate the AoA [9]. To evaluate angle error profile, an experiment with a 2.45 GHz two-antenna array (with 6.1 cm distance between antennas) is placed 5 meters in LOS from the RF signal generator (Figure 8a). The signal generator outputs a 10 dBm signal at 2.45 GHz. The antenna array is then rotated around its center by 90° (in 5° steps). The rotation is reflected in phase difference from 0 to 180°. For every angle step, the output voltage is observed and mapped to the phase difference Figure 9. The difference between the actual and the measured phase differences is then plotted in Figure 10a which is close to the reported phase error profile in [9] for 2.2 GHZ (shown in Figure 10b).

### 5.2. Polynomial Regression of an Empirical Non-Linear AoA Error 

The AD8302 is a fully integrated system for measuring gain/loss and phase in numerous receive, transmit, and instrumentation applications [9]. The AD8302 includes a phase detector with a precise phase balance. An example of the performance of the AD8302 over 900 MHz is shown in Figure 8. Note that the phase measurement accuracy is high over a wide-angle range (i.e., the phase difference error is less than 0.3° from 20° to 160°). However, for instance, the error for a phase difference φ above 160° is growing rapidly from 0.3° until it reaches 9° at 180° phase difference. The same trend is noticed at phase differences below 20°. Therefore, as the phase error is not uniform, polynomial regression is applied to approximate the empirical phase error reported in AD8302 at 900 MHz.

**Figure 11:**
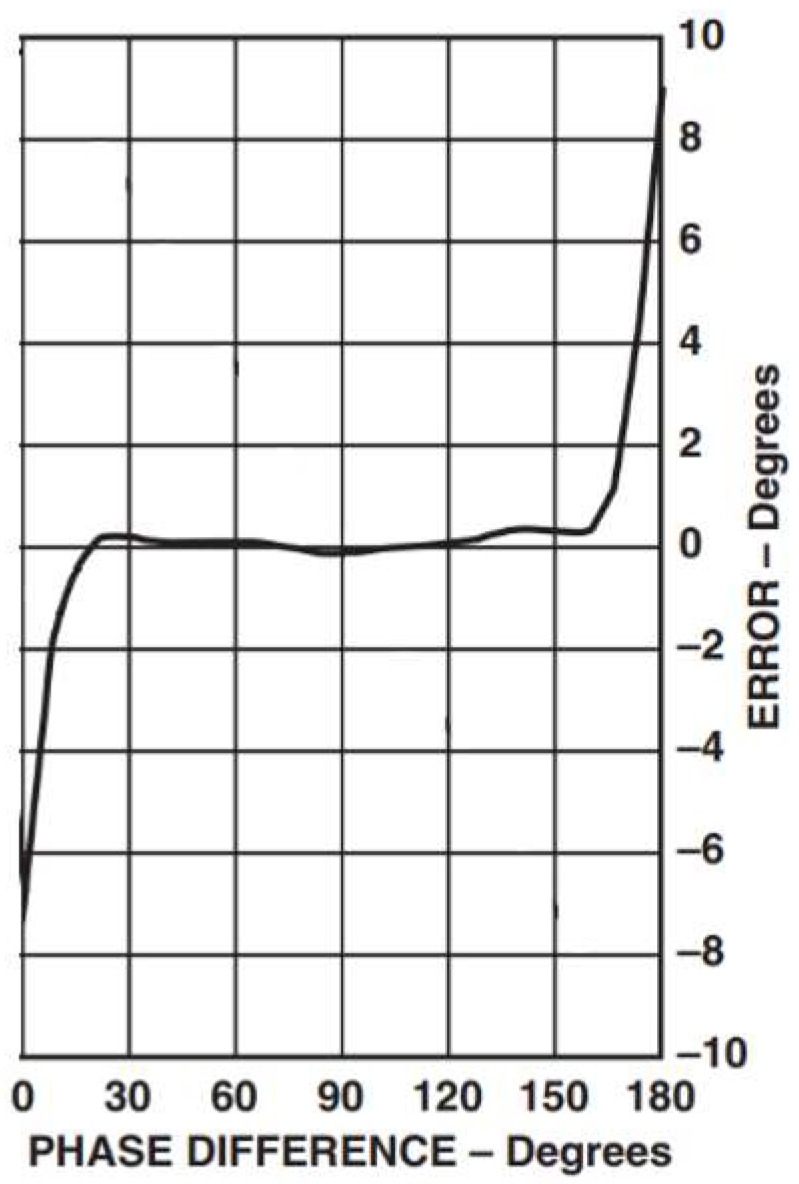
Empirical error profile of the phase difference AD8302 for 900 MHz [9].

The resultant polynomial regression of the phase error in degrees (shown in Figure 11) is given by Equation (4) below. The measured error at high AoAs (vertical or horizontal) is always positive (i.e., large angles is underestimated). However, for low AoAs (vertical or horizontal), the error is negative (i.e., small angles are overestimated). Therefore, vertical or horizontal θ in Figure 3 is equal to $\pi -\varphi -\frac{Error}{2}$, with an error margin of $\pm \varepsilon =\;\pm \frac{Error}{2}$.

(4) $$Error\;=\;\{\begin{array}{c} 4.41*10^{-6}\varphi^{4}-2.12*10^{-4}\varphi^{3}-7.96*10^{-3}\varphi^{2}+0.57\varphi^{1}-7.15\;0{}^{\circ}\leq \varphi \leq 45{}^{\circ}\;\\ 2.04*10^{-22}\varphi^{4}-5.74*10^{-20}\varphi^{3}+5.86*10^{-18}\varphi^{2}-2.56*10^{-16}\varphi^{1}+0.09\;45{}^{\circ}<\varphi \leq 135{}^{\circ}\\ 2.79*10^{-6}\varphi^{4}-1.51*10^{-3}\varphi^{3}+0.31\varphi^{2}-27.07\varphi^{1}+893.25\;135{}^{\circ}<\varphi \leq 180{}^{\circ} \end{array},$$

### 5.3. Simulation Setup and Results

**Figure 12:**
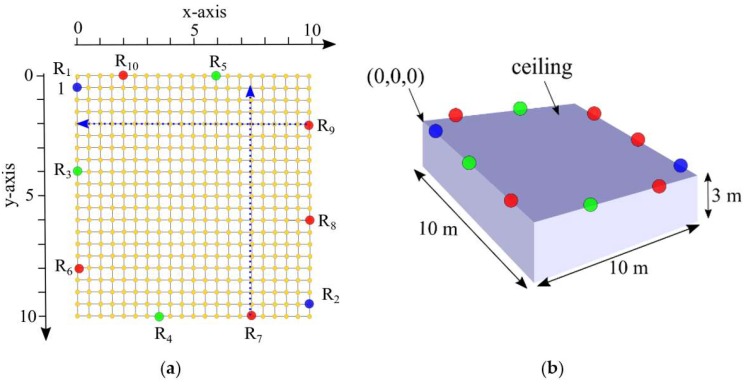
(**a**) Top view the grid and reader placement. blue lines represents examples of tag locations where $\theta_{h}\;=\;\pi \;$; the readers are placed in a non-collinear positions (i.e., no blue lines overlap) (**b**) 3D illustration of the reader placement at the ceiling of the localization area.

In our numerical evaluation of the proposed scheme, 2, 5, and 10 readers are placed at an average height of 3 m alongside the ceiling edge or at the sidewalls of a 10 m × 10 m room as shown in Figure 12. Grid points are placed on the floor of the room with a grid width of 0.5 m. To test the performance of the proposed TDEAL technique, a tag is placed on all grid points of the room at heights of 0, 1.5, 2.5, and 3 m. The readers’ positions are mapped to the three-dimensional Cartesian coordinate system.

**Figure 13:**
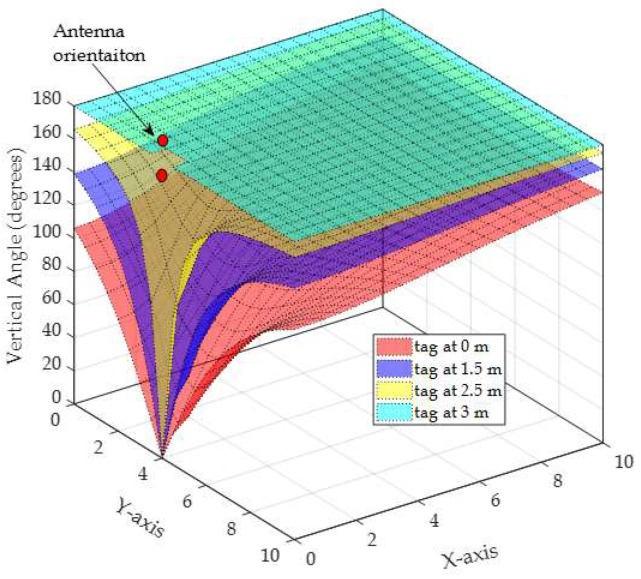
Actual vertical phase difference for a tag at all *x*-*y* grid locations with different heights of 0, 1.5, 2.5, and 3 m. The vertical antenna is centered at (0,4,3) in the *x*, *y*, *z*-space.

**Figure 14:**
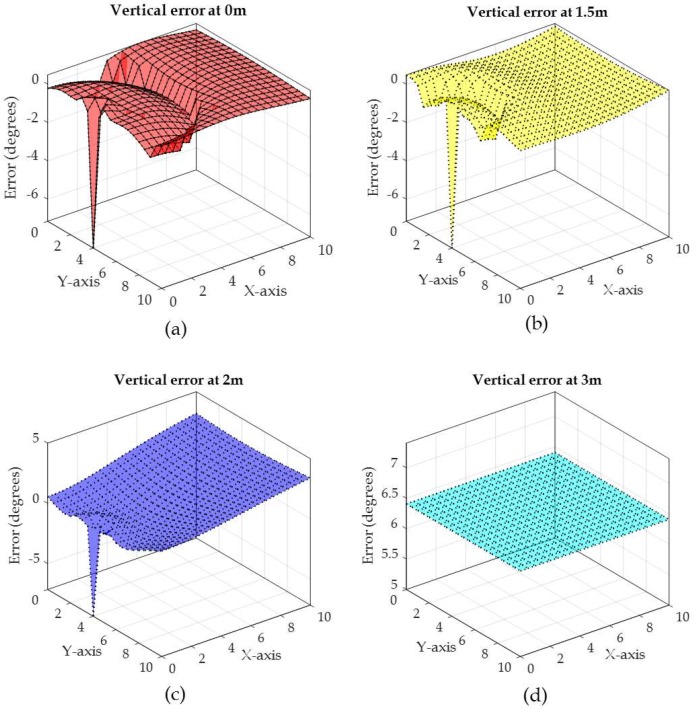
Phase difference error for a tag at all *x*-*y* grid locations with different heights of: (**a**) 0 m, (**b**) 1.5 m, (**c**) 2.5 m, and (**d**) 3 m.

**Figure 15:**
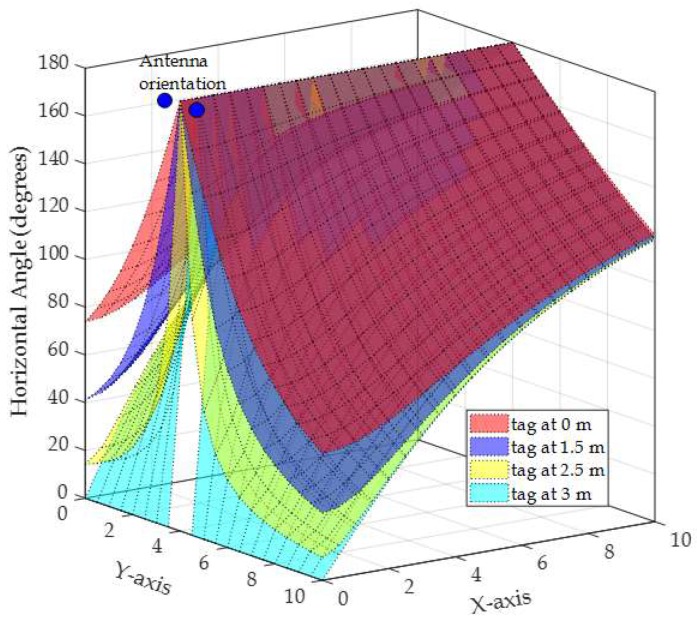
Actual horizontal phase difference for a tag at all *x*-*y* grid locations with different heights of 0, 1.5, 2.5, and 3 m. The horizontal antenna is centered at (0, 4, 3) in the *x*, *y*, *z*-space.

**Figure 16:**
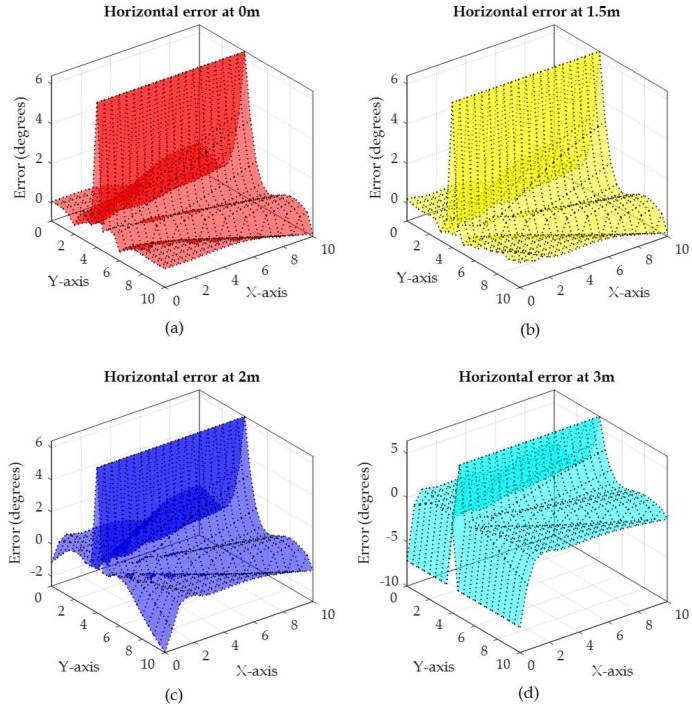
Horizontal phase difference error for a tag at all *x*-*y* grid locations with different heights of: (**a**) 0 m, (**b**) 1.5 m, (**c**) 2.5 m, and (**d**) 3 m.

To illustrate the relation between the measured angle from vertical and horizontal antenna arrays on the error in the phase difference, an example of the actual vertical angle ($\theta_{v}$) and horizontal angle ($\theta_{h}$) along with the vertical angle error and the horizontal angle error are depicted in Figure 13, Figure 14, Figure 15 and Figure 16, respectively. The vertical and horizontal phase differences, $\varphi_{v}$ and $\varphi_{h}$, are measured by the vertical and horizontal antenna arrays of R3 (with *x*,*y*,*z*-coordinates of (0,4,3) in Figure 12) from a tag at all grid locations with heights of 0, 1.5, 2.5, and 3 m for 900 MHz communication frequency. The tag’s *x*-*y* location on the grid and its height dictate the phase difference φ (recall that θ = π−φ) as shown in Figure 13 and Figure 15. The phase difference is then mapped to the empirical phase differences error profile as shown in Figure 14 and Figure 16.

In Figure 13, the vertical phase differences are mostly between 45° and 135° when the tag is at the heights of 0 and 1.5 m. This is reflected in low angle error as shown in Figure 14a,b as the error profile within this range of angles is low (as in Figure 11). At tag heights of 2.5 and 3 m, the phase differences in Figure 13 are mostly higher than 135°, which are reflected in higher phase difference error as shown in Figure 14c,d. In fact, at the height of 3 m, $\theta_{v}$ is constant and equals 180° which is mapped to a constant error around 6.5°. In Figure 13, when the tag’s *x*,*y*-coordinates are closer to the reader’s *x*,*y*-coordinates (i.e., (*x* = 0, *y* = 4)), $\theta_{v}$ is low and around 0°, which is also reflected in high angle error (around −7°).

In Figure 15, the horizontal phase differences are mostly within the phase difference between 40° and 180° when the tag is at the heights of 0 and 1.5 m. This is reflected in low angle error as shown in Figure 16a,b as the error profile within this range of angles is low (as in Figure 11). Note that in Figure 15, regardless of the tag height, when the tag’s *y*-coordinates are closer to the reader’s *y*-coordinates (i.e., 4), $\theta_{h}$ is high (around 180°), which is reflected in a high angle error of around 6.5° as shown in Figure 16a–d. At tag heights of 2.5 and 3 m, the phase difference in Figure 15 ranges from 0° to 180°. This is reflected in higher phase difference error as shown in Figure 16c,d. In fact, at a height of 3 m $\theta_{h}$ is around 0° when the tag *x*-coordinates are closer to the reader’s *x*-coordinates (i.e., *x* = 0) which is mapped to a constant error around −6.5°. As the phase difference defines the difference slope, high error angles will produce high error location estimation, as will be discussed shortly in the following two evaluation scenarios.

In summary, Within the localization area, $\theta_{v}$ and $\theta_{h}$ will always be less than π and more than 0 (refer to Figure 13, Figure 14, Figure 15 and Figure 16) unless:

1. The tag is at the same *x*,*y*-coordinates of the reader (reflected in $\theta_{v}\;=\;0{}^{\circ}$,$\theta_{h}\;=\;180{}^{\circ}$).
2. The tag is at the same elevation of the reader (reflected in $\theta_{v\;}=\;180{}^{\circ}$).
3. The tag is at the same *x*- or *y*-coordinates if the reader is mounted on walls parallel to the *x*- or *y*-axis, respectively (reflected in $\theta_{h}\;=\;180{}^{\circ}$).
4. The tag is at the same height of the reader and the same *x*- or *y*-coordinates, if the reader is mounted on walls parallel to the *x*- or *y*-axis, respectively (reflected in $\theta_{h\;}=\;0{}^{\circ}$).

In the following, we evaluate TDEAL with two scenarios. The *z*-coordinate (height) of the readers is set to (a) same height of 3 m in evaluation scenario 1, (b) different heights that are uniformly distributed from 2.55 to 3.45 m in evaluation scenario 2.

**Figure 17:**
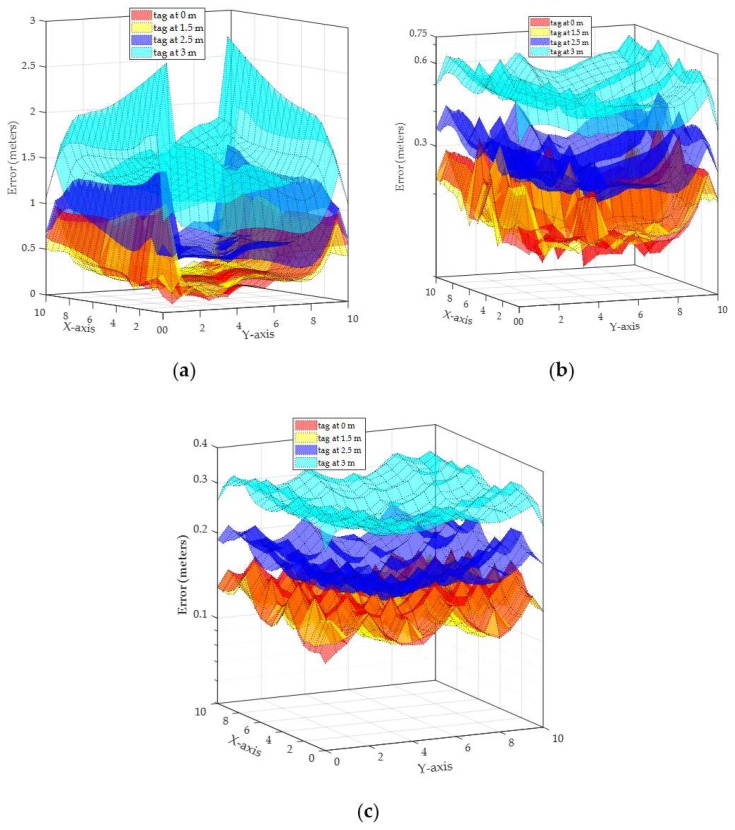
Localization error for tags at 0, 1.5, 2.5, and 3 m for 900 MHz antenna array with all readers at the same height of 3 m. (**a**) Localization error by 2 readers, (**b**) localization error by 5 readers, (**c**) localization error by 10 readers.

*Scenario 1* (all readers at the same height): the localization error between the actual tag location and the estimated tag location by TDEAL technique is reported. The empirical phase error of AD8302 at 900 MHz in Equation (4) is applied by all readers to calculate AoA. The localization error of a tag that is detected by the two readers $R_{1}$ and $R_{2}$ (blue circles in Figure 12) is plotted in Figure 17a for a tag at heights of 0, 1.5, 2.5, and 3 m from the floor. Similarly, the localization error of a tag by 5 readers, $R_{1},R_{2},R_{3},R_{4},$ and $R_{5}$ in Figure 12, for a tag at the different heights in 0, 1.5, 2.5, and 3 m is plotted in Figure 17b. In Figure 17c, localization error is plotted for a tag that is detected by all readers in Figure 12.

The reported results in Figure 17 provides a comprehensive overview of the localization performance of TDEAL.

The first observation is the error dependency on the tag’s height. The lower the tag’s height, the higher the accuracy (i.e., average error at height 0 m is lower than the average error at height 1.5 m and above as shown in Table 1). Based on the 900 MHz empirical error profile, the margin between 20° and 160° has a relatively low phase error. Therefore, the following can be concluded:

(1) For the same number of localizing readers, the localization accuracy gets higher as the elevation of the tags gets lower (closer to the floor).
(2) A dramatic increase in the mean location error is observed when the tag is at the same elevation of the localizing readers due to the high error in $\theta_{v}$ at all readers.

The second observation describes the error dependency on the number of readers. From Figure 17, it is clear that higher number of readers yield more estimation points (${\hat{F}}_{k})$. TDEAL with its corresponding angle-distance based weights increases the accuracy by damping the estimations that are based on high angle or distance error.

The third observation in Figure 17 is the comparable localization error for a tag at an elevation of 0 and 1.5 m from the floor. At 0 m elevation, the tag has a lower average error in $\theta_{v}$. However, recall that the error weight is defined by both the error in AoA and the distance from the localizing pair of readers. The average distance from the reader at an elevation of 0 m is larger than the average distance at 1.5 m. This angle–distance tradeoff causes such comparable average localization error.

*Scenario 2* (readers at different heights): In this scenario, we evaluate the localization accuracy when the readers are not at the same height from the floor. To maintain the applicability of the TDEAL algorithm, the lowest reader is placed at a height above the maximum height of the tag. This is justifiable as in indoor environments the tags are usually are attached to humans or mobile objects with known maximum possible height.

**Figure 18:**
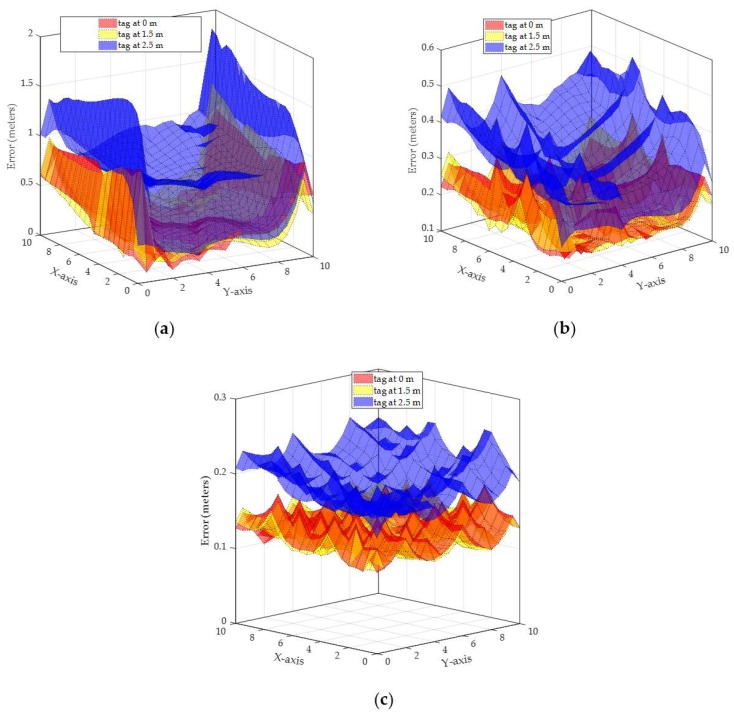
Localization error for tags at 0, 1.5, 2.5, and 3 m for 900 MHz antenna array with all readers at different heights. (**a**) Localization error by 2 readers, (**b**) localization error by 5 readers, (**c**) localization error by 10 readers.

In this scenario, the reader $R_{1}$ in Figure 12a is placed at the sidewalls with heights: $R_{1}=2.55,\;R_{2}\;=\;3.45,\;R_{3}\;=\;2.65,\;R_{4}=\;3.35,\;R_{5}=\;3.05,\;R_{6}=\;2.95,\;R_{7}=\;2.75,\;R_{8}=\;3.25,\;R_{9}=\;2.85,\;R_{10}=\;3.15$ m. As the average height of the readers in the first scenario was 3 m; the readers in the second scenario are also at an average height of 3 m. Therefore, as the lowest reader is placed at 2.55 m, this scenario is evaluated for tags heights of 0, 1.5, and 2.5 m as shown in Figure 18 and Table 1. Similar to the plot in Figure 17, errors at each tag location with a height of 0, 1.5, and 2.5 m are plotted in Figure 18 for a tag that is detected by 2, 5, and 10 readers, respectively. The simulated localization error trends are similar to that observed in the result in Figure 17. However, the differences between the results in Figure 17 and Figure 18 are caused by having readers at heights that are higher and lower than the average readers’ height.

The readers above the average readers’ height (i.e., above 3 m), have lower average phase difference errors, especially vertical phase difference error (as shown in Figure 14a,b). Note that higher readers have higher distance to the tag which is reflected in lower weight for the readings from such readers. On the other hand, readers that are lower than the average readers’ height, have higher phase difference error in both vertical and horizontal phase difference errors (as shown in Figure 14c,d and Figure 16c,d). The results in Figure 18 are comparable to the results shown in Figure 17 and both are summarized in Table 1.

TDEAL is also simulated with readers at the same height and with readers at different heights for two error profiles of 2.45GHz communication channels. The results for both 900 MHz and 2.45 GHz error profiles are presented in Table 1. Note that the average localization error by TDEAL is not significantly affected by the readers’ heights unless the number of readers is small and their height is close to the tag’s height. The difference in average error noted by asterisks in Table 1 is caused by having $R_{1}$ at 2.55 m which produces high angle errors when the tag height is 2.5 m (as in Figure 18a). It can be concluded that different readers heights (with a given average height) do not affect the localization accuracy when compared to the results form readers at the same height. On the other hand, the localization error is affected by the angle error profile (i.e., communication frequency). However, the TDEAL algorithm is capable of impeding the effect of angle error profiles when the tag is detected by more than two readers (i.e., 5 and 10 readers).

## 6. Conclusions

In this paper, the TDEAL technique for localizing tags under empirical AoA error is proposed. TDEAL utilizes at least two readers with vertical and horizontal antenna arrays to estimate the tag location. The proposed technique simplifies hyperbola equations to a less complicated solution that is based on the hyperbola’s asymptote equations (cones equations in three-dimensional space) which yields vectors instead of hyperbola intersection trajectories. TDEAL is designed so that two non-collinear readers measure the vertical and horizontal AoA angles from a given tag and report them to a central database. Then the tags’ locations are estimated from all combinations of any two readers while accounting for the inherited angle error in such a location estimate. Angle- and distance-dependent weights are assigned by the proposed technique to hinder high error-location estimates.

The simulation of TDEAL based on the polynomial approximation of the empirical phase error profile validates the effectiveness of the proposed TDEAL under realistic assumptions. It is shown that the TDEAL technique is capable of accurately estimating the location of tags (at different heights) in a relatively large localization area. For instance, with 10 readers TDEAL was able to achieve an average error of less than 13 cm with a tag height of 1.5 m. The integration between TDEAL and range-free localization or inertial navigation systems is a future direction to improve localization accuracy under the constraint of a low number of readers.

In the future, we are planning to extend this work to evaluate TDEAL under NLOS conditions. We also look forward to implementing TDEAL for tracking applications in which subsequent location estimation (e.g., through Kalman filter) can be applied for the targeted indoor applications. The potential optimizing of the readers’ location as a function of the empirical error profile is also part of our future plans.

## Figures and Tables

**Table 1:** Summarizing statistics of the different simulated localization scenarios.

			900 MHz				2.45 GHz			
			Tag Height (meters)				Tag Height (meters)			
			0	1.5	2.5	3	0	1.5	2.5	3
Readers at the same height	2 readers	Average error (m)	0.33	0.33	0.66	1.44	0.57	0.87	1.79	2.96
		Maximum error (m)	1.09	1.19	1.53	2.72	2.20	2.82	3.48	5.75
	5 readers	Average error (m)	0.20	0.19	0.30	0.52	0.29	0.37	0.66	0.99
		Maximum error (m)	0.37	0.36	0.47	0.73	0.64	0.74	1.03	1.40
	10 readers	Average error (m)	0.12	0.13	0.18	0.28	0.18	0.22	0.37	0.52
		Maximum error (m)	0.18	0.18	0.24	0.34	0.26	0.33	0.46	0.65
Readers at different heights	2 readers	Average error (m)	0.33	0.36	0.87	-	0.58	0.92	2.02	-
		Maximum error (m)	1.10	1.32	1.85	-	2.20	2.96	4.27	-
	5 readers	Average error (m)	0.20	0.20	0.33	-	0.29	0.38	0.70	-
		Maximum error (m)	0.38	0.38	0.53	-	0.65	0.78	1.07	-
	10 readers	Average error (m)	0.13	0.13	0.19	-	0.18	0.23	0.38	-
		Maximum error (m)	0.18	0.18	0.25	-	0.26	0.33	0.49	-
